# Genome-wide identification, evolution and expression profiles analysis of bHLH gene family in *Castanea mollissima*

**DOI:** 10.3389/fgene.2023.1193953

**Published:** 2023-05-12

**Authors:** Liyang Yu, Cao Fei, Dongsheng Wang, Ruimin Huang, Wang Xuan, Chunlei Guo, Liu Jing, Wang Meng, Lu Yi, Haie Zhang, Jingzheng Zhang

**Affiliations:** ^1^ Engineering Research Center of Chestnut Industry Technology, Ministry of Education, Hebei Normal University of Science and Technology, Qinhuangdao, Hebei, China; ^2^ Hebei Collaborative Innovation Center of Chestnut Industry, Qinhuangdao, Hebei, China; ^3^ Hebei Key Laboratory of Horticultural Germplasm Excavation and Innovative Utilization, Qinhuangdao, Hebei, China

**Keywords:** *Castanea mollissima*, bHLH family, duplication model, expression pattern, collinearity analysis

## Abstract

The basic helix-loop-helix (bHLH) transcription factors (TFs) gene family is an important gene family in plants, and participates in regulation of plant apical meristem growth, metabolic regulation and stress resistance. However, its characteristics and potential functions have not been studied in chestnut (*Castanea mollissima*), an important nut with high ecological and economic value. In the present study, 94 *CmbHLHs* were identified in chestnut genome, of which 88 were unevenly distributed on chromosomes, and other six were located on five unanchored scaffolds. Almost all CmbHLH proteins were predicted in the nucleus, and subcellular localization demonstrated the correctness of the above predictions. Based on the phylogenetic analysis, all of the *CmbHLH* genes were divided into 19 subgroups with distinct features. Abundant *cis*-acting regulatory elements related to endosperm expression, meristem expression, and responses to gibberellin (GA) and auxin were identified in the upstream sequences of *CmbHLH* genes. This indicates that these genes may have potential functions in the morphogenesis of chestnut. Comparative genome analysis showed that dispersed duplication was the main driving force for the expansion of the *CmbHLH* gene family inferred to have evolved through purifying selection. Transcriptome analysis and qRT-PCR experiments showed that the expression patterns of *CmbHLHs* were different in different chestnut tissues, and revealed some members may have potential functions in chestnut buds, nuts, fertile/abortive ovules development. The results from this study will be helpful to understand the characteristics and potential functions of the bHLH gene family in chestnut.

## Introduction

Transcription factors (TFs) are a class of functional protein factors widely existing in eukaryotes, and they regulate the expression of downstream genes through specific binding with *cis*-acting elements of DNA, and participate in many biological processes (Yu et al., 2023). Basic helix-loop-helix protein (bHLH) transcription factors widely exist in eukaryotes and their member number next only to the MYB family, whose name was derived from their specific protein structure contained basic amino acid regions and helix-loop-helix regions (Ledent and Vervoort, 2001; Baudry et al., 2006). The *bHLH* genes mostly exist in the form of gene family in plant genome, and have important functions in plant signal transduction, regulation of plant apical meristem growth, and stress resistance (Bailey et al., 2003; Komatsu et al., 2003). The bHLH gene family has been analyzed in multiple plants, such as *Arabidopsis thaliana* (Bailey et al., 2003), rice (*Oryza sativa*) (Li et al., 2006) and tomato (*Solanum lycopersicum*) (Sun et al., 2015; Wang et al., 2015), and the *bHLH* genes in this three model plant species were divided into subgroups 21, 22 and 24, respectively, which means that members of the bHLH gene family have different subgroups in other plants (Pires and Dolan, 2010b).

The functions of the *bHLH* genes in model plants have been widely characterized, participating in multiple biological processes such as morphogenesis, stress resistance, signal transduction and secondary metabolism (Li et al., 2006). For example, *PIF4* plays a role in the elongation of the hypocotyl of *A. thaliana* by participating in phyB-regulated photomorphogenesis (Huq and Quail, 2002). During the development of *A. thaliana* embryo, the expression of *RGE1* gene in endosperm can regulate the growth of embryo (Kondou et al., 2008). *AtbHLH112* has a positive regulatory effect on salt resistance, drought resistance and osmotic resistance, but inhibits root development (Yujia et al., 2015). *OsbHLH148*, a *bHLH* gene in rice, constitutes the initial response of the OsbHLH148-OsJAZ-OsCOI1 signal module in rice to the expression of drought resistance genes regulated by jasmonic acid (JAs) (Seo et al., 2010). *SlbHLH59* is directly bound to the *PMM*, *GMP2* and *GMP3* gene promoters in the D-mannose/L-galactose biosynthesis pathway, one of the ascorbic acid biosynthesis pathways, to promote the accumulation of ascorbic acid in tomato (Ye et al., 2019). Overexpression of *ZmPTF1* in maize (*Zea mays*) can promote root cap development and increase biomass under low phosphorus stress (Li et al., 2011). However, the potential functions of *bHLH* genes in chestnut are still unclear.

Chestnut is one of the earliest domesticated plants in ancient times, with a cultivation history of more than 6,000 years in China, which is widely distributed in the northern hemisphere due to its good adaptability (Hu et al., 2022; Yu et al., 2023). Chestnut is not only an ecological tree species, but also has nutritional value. The worldwide chestnut production is about 2.27 million tons (Food and Agriculture Organization of the United Nations), and this number has been increasing continuously for more than 10 years, which indicates that consumers have great confidence in the health of chestnut nut (Massantini et al., 2021). In addition to being a good source of starch, chestnut nut is also rich in a variety of sugars, vitamins, and other biological active substances and minerals required by human body (Hu et al., 2022; Yu et al., 2023). Notably, more and more attention has been paid to the medicinal value of chestnut nut. The proper amylose/amylopectin ratio in chestnut nut has attracted attention as a preventive factor against diabetes and allergy (Choupina, 2019). Some terpenoids in chestnut nut have effects on inhibiting the expansion of HeLa tumor cells (Gao et al., 2010). Chestnut nut is rich in antioxidant substances such as ascorbic acid, phenols and flavonoids, which are very beneficial to human antibacterial and anti-cancer (Abe-Matsumoto et al., 2010; Dinis et al., 2012; Chang et al., 2020; Xu et al., 2020). In fact, the research on the antiviral effects, common disease prevention and anti-aging of chestnut nut has been carried out, which undoubtedly further increases the potential utilization value of chestnut nut (Kawasaki et al., 2009; Lupini et al., 2009; Noh et al., 2010). However, as a monoecious plant, the proportion of female and male flowers in chestnut can reach 1:2,400–4,000, and the low number of female flowers and excessive consumption of tree nutrition by abundant male flowers are significant causes for limiting the yield of chestnut (Chen et al., 2019; Yu et al., 2023). One of the important functions of the ovule is to promote the normal growth and development of flowers, and the ovule is the female reproductive organ of flower and an important part of sexual reproduction (Gómez et al., 2019). Furthermore, excavating genes that regulate the accumulation of bioactive substances in chestnut nut can be used as the first step in cultivating new high-quality chestnut varieties (Hu et al., 2022). Notably, the functions of *bHLH* genes in plants are diversified, such as GA signal transduction regulation, meristem growth and fruit development, and these are all related to factors that limit the development of the chestnut industry, such as imbalanced male and female flowers, poor nut quality (Sun et al., 2020; Yu et al., 2023). Thus, it is very significant to excavate the genes that regulate the fertility of chestnut ovules and nut development. The publication of the whole genome sequence of chestnut has enabled us to excavate the *bHLH* genes which potentially regulate growth and development by comprehensively studying the gene structure and evolutionary characteristics of bHLH gene family (Wang et al., 2020).

Chestnut is an excellent tree species with nutritional, medicinal and ecological values, and the extremely small ratio of female to male flowers (1:2,400–4,000) is an important reason for limiting chestnut yield (Chen et al., 2019; Yu et al., 2023). Studies have confirmed that bHLH factors play an important role in the development of ovule, which is an important part of sexual reproduction and promote the normal development of flowers (Heisler et al., 2001; Huq and Quail, 2002). Whether *bHLH* genes play a role in chestnut development has not been reported. Here, the bHLH gene family in chestnut has been identified and characterized first time, with analyzing the physicochemical properties, phylogenetic comparison, gene structure, *cis*-acting elements and duplication model. Moreover, we also compared the expression patterns of all identified *bHLH* genes in chestnut buds, nuts, fertile/sterile ovules at different development stages. To verify the above analysis, several *bHLH* genes were validated using qRT-PCR and subcellular localization assays. This study provided a foundation for further analysis of the potential mechanism of *bHLH* genes in chestnut growth and development, especially for aiming to identify candidate genes that may be involved in the development of chestnut buds, nuts, fertile/sterile ovules.

## Materials and methods

### Gene identification, enrichment and subcellular location analysis

The published 158 *bHLH* genes in *A. thaliana* were used as a query sequence to run the BLAST (Basic Local Alignment Search Tool) program on the protein dataset of chestnut with default parameters. Furthermore, we used the latest Hidden Markov Model (HMM) file of the bHLH domain (PF00010) to search against the all proteins of chestnut using HMMER 3.0 (Evalue < 1e-5). After determine the existence of the bHLH domain using the NCBI Batch CD Search tool, we obtained 94 *bHLH* genes in chestnut, which were renamed *CmbHLH1* to *CmbHLH94* based their order on chromosome. The ExPasy website was used to obtain their physicochemical properties, and we used the eggNOG-mapper (Cantalapiedra et al., 2021) to obtain GO and KEGG annotation. We performed GO and KEGG enrichment analysis and visualization using TBtools (Chen et al., 2020). The subcellular localization of *CmbHLH* genes were predicted by using the online tool Cell-Ploc (Chou and Shen, 2010), and the full length coding sequence (CDS) without stop codon was cloned into the pAN580 and N-terminal fused with the green fluorescent protein (GFP) to verify the location of *CmbHLH9*, *CmbHLH25* and *CmbHLH55*. The fusion constructs then transiently expressed into *A. thaliana* protoplasts.

### Phylogenetic and sequence analysis

MEGA 7.0 (Kumar et al., 2016) was used to construct a phylogenetic tree for the *bHLH* genes in chestnut with the maximum likelihood method, and we used the “Find Best DNA/Protein Models (ML)” function to find the best amino acid substitution model (partial deletion 95%). The final parameters were as follows: Jones–Taylor–Thornton (JTT) model; Gamma Distributed (G); Partial deletion 95%; 1,000 bootstrap replications. A phylogenetic tree of *CmbHLH* genes was constructed with neighbor-joining method using MEGA 7.0 with parameters: Poisson model, pairwise deletion, and 1,000 bootstrap replications. Additionally, Bayesian method was used to construct a phylogenetic tree of *CmbHLHs*. Briefly, MAFFT 7.0 (Katoh and Standley, 2013) and ModelFinder (Kalyaanamoorthy et al., 2017) were used to perform multiple sequence alignments and select optimum protein substitution model, respectively. MrBayes 3.2 (Ronquist et al., 2012) was used to construct Bayesian tree based on the best-fit model: JTT + F + I + G4. TBtools was used to visualize the structure of *CmbHLH* genes. The conserved domain and motif information of the *CmbHLH* genes were obtained from the Batch-CDD and MEME online programs, respectively. The combined analysis of phylogenetic tree, conserved domain and motif distribution was used to explore the sequence characteristics in different subgroups. The psRNATarget (Dai and Zhao, 2011) and Simple Sequence Repeat Identification Tool (SSRIT) on Gramene website (Tello-Ruiz et al., 2021) were used to predict miRNA targets and SSRs of the *CmbHLH* genes, respectively. The 3D structure models of all CmbHLH proteins were predicted through Swiss Model.

### Collinearity analysis of *CmbHLH* genes

To further explore the evolution of the *CmbHLH* genes, the Multiple Collinearity Scan Toolkit (MCScanX) program (Wang et al., 2012) was used to perform a collinearity analysis within chestnut and with *A. thaliana*, rice, oak and grape genomes. Notably, we used a detailed method as in our previous study to determine the duplication model of the *CmbHLH* genes (Yu et al., 2022a). Based on the algorithm of the MCScanX program (Wang et al., 2012), the protein-coding genes from chestnut genome was compared against itself, and the duplicate genes were first defined as i) “dispersed duplicates”; ii) If the distance or gene rank between two duplicate genes is less than 20, they are re-labeled as “proximal duplicates”; iii) If the duplicate genes are adjacent (gene rank is 1), they are re-labeled as “tandem duplicates”; iv) The duplicated genes in the collinearity regions are re-labeled as “WGD or segmental ". The MCScanX program was run to directly obtain *CmbHLH* genes from proximal, dispersed and tandem duplication. Specifically, based on the collinearity results within the chestnut genome, we drawn the homologous collinearity dot-plot within the chestnut genome. Further, non-synonymous (Ka) and synonymous substitution sites (Ks) values of homologous gene pairs on the homologous collinear blocks were calculated by KaKs_Calculator (Wang et al., 2010), and the median Ks values was calculated by writing script (Yu et al., 2023). The median Ks values of homologous collinear blocks containing the *CmbHLH* genes were labeled on the dot-plots. Finally, based on the distribution of Ks values corresponding to the whole genome duplication (WGD) event that occurred in the chestnut genome in our earlier stage (Yu et al., 2023), combined with the complementarity of homologous collinear blocks, we finally determined the *CmbHLH* genes formed by WGD event.

### *Cis*-acting elements analysis

Based on the structure annotation of the chestnut genome, the 2000 bp upstream sequences of 94 *CmbHLH* genes were extracted from the chestnut genome sequence. The upstream sequences of *CmbHLH* genes were submitted to PlantCARE software for identification of *cis*-acting elements and function categorization. We used Tbtools to visualize the predicted *cis*-acting elements, except for those members without specific function.

### Expression analysis of *CmbHLH* genes

In order to explore the potential function of the *CmbHLH* genes in different tissues of chestnut, public database resources were utilized. RNA-seq data of chestnut buds at different stages (20, 25 and 30 days post-anthesis), chestnut nuts at different stages (70, 82 and 94 days post-anthesis), chestnut fertile/abortive ovules at different stages (15-July, 20-July and 25-July) were obtained from NCBI, and corresponding accession numbers were in Supplementary Table S1. The above transcriptome data from chestnut buds, nuts and fertile/abortive ovules were all three biological replicates. All RNA-seq reads were mapped on the reference genome (N11-1 from Castanea Genome Database) and the Kallisto software was used to quantify transcriptome data into Transcripts Per Kilobase of exon model per Million mapped reads (TPM). Tbtools was used to map the heatmaps for exhibiting the expression level of *CmbHLH* genes. The “Normalized” (scale method) in TBtools was used to normalize the expression, and the heatmaps were created by TBtools based on the transformed data of log_2_ (TPM + 1) values.

### Plant material and qRT-PCR analysis

The samples used for real-time quantitative PCR experiments were the nuts of “Yan Long” chestnut planted by Hebei Normal University of Science and Technology at 70, 82, and 94 days post-anthesis with three biological replicates. All samples were immediately frozen in liquid nitrogen and stored at −80°C for RNA extraction. The HiPure Total RNA kit (Magen, R4111, China) was used to extracted and isolated total RNA. PrimeScript RT Master Mix (Takara Biotechnology Co., Dalian, China) was used to reverse transcribe RNA into single stranded cDNA. Real-time quantitative PCR experiments were performed on ABI 7500 Real-Time PCR system (Applied Biosystems Inc., Foster City, CA, United States) with TB Green Premix Ex Taq (Takara). The relative gene expression values were calculated using the comparative CT (2^-△△CT^) method (Livak and Schmittgen, 2001), and *18S* gene of chestnut was used an internal control.

## Results

### Identification, physicochemical properties, classification and function annotation

**FIGURE 1:**
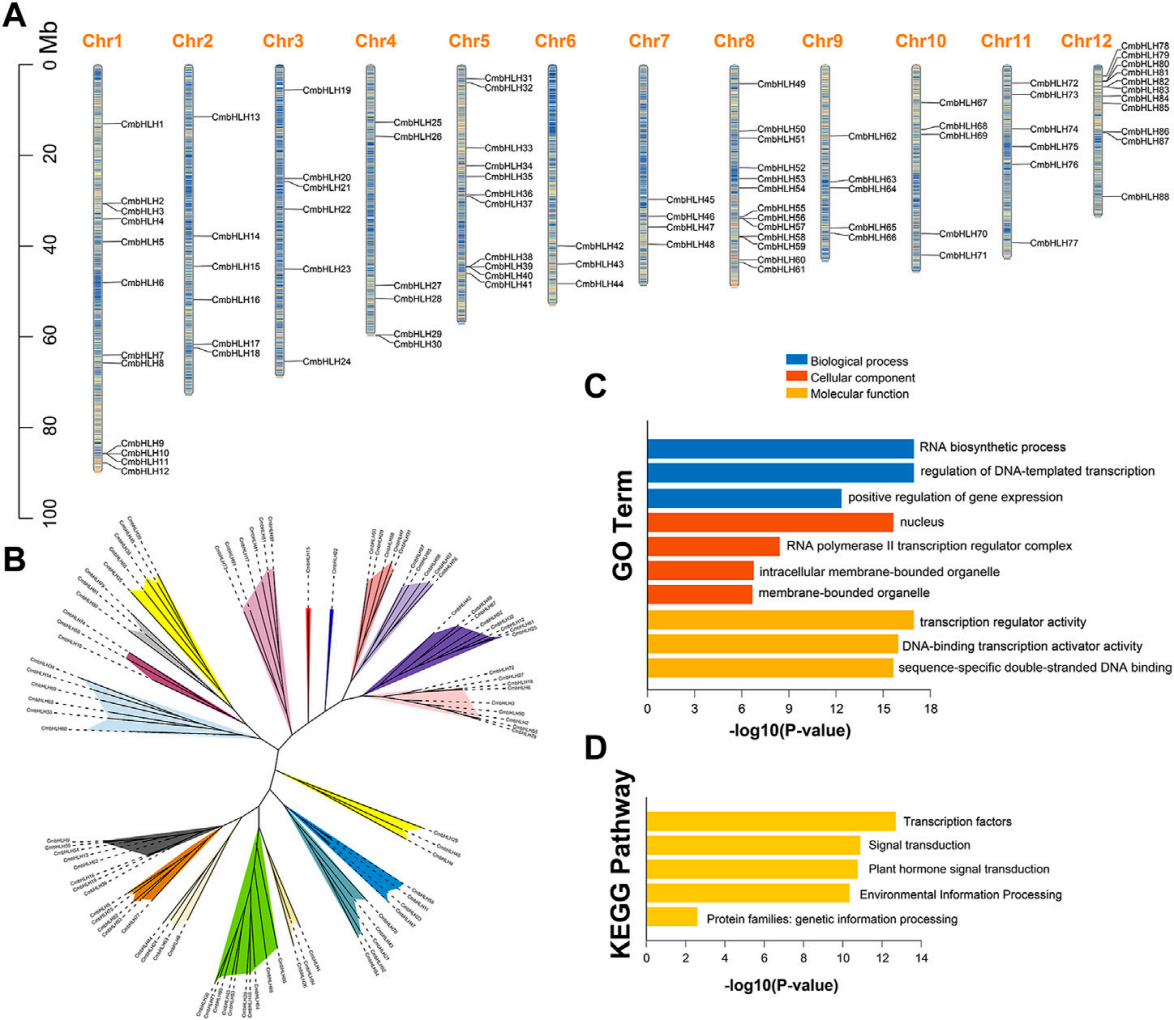
Chromosome distribution, phylogenetic analysis and functional enrichment analysis of the *CmbHLH* genes. **(A)** Chromosome distribution of *CmbHLH* genes. The color of segments in the chromosomes shows the gene density of the corresponding region. **(B)** Unrooted phylogenetic tree of CmbHLH proteins. MEGA 7.0 was used to construct the phylogenetic tree based on the protein sequences with maximum likelihood method. The proteins were clustered into 19 groups. Different background colors indicate the different group of the CmbHLH proteins. **(C)** GO function enrichment analysis of *CmbHLH* genes. **(D)** KEGG function enrichment analysis of *CmbHLH* genes.

We identified 94 *bHLH* genes in chestnut genome using the BLASTP and HMMER 3.0 programs, and they were designated as *CmbHLH1* to *CmbHLH94* based on their relative position on chromosome and scaffold (Figure 1A; Supplementary Table S2). Eighty-eight *CmbHLH* genes were randomly distributed on all 12 chromosomes and other six *CmbHLH* genes were located on five unanchored scaffolds. Chromosome 8 had the highest number of *CmbHLH* members (13), while chromosome 6 had the fewest *CmbHLH* members (3) (Figure 1A). The analysis of all CmbHLH protein sequences showed that they had extensive variation in physicochemical properties (Supplementary Table S2). The length of CmbHLH proteins varied from 159 to 711 aa, molecular weight ranged from18.0 to 79.2 KDa. Most CmbHLH proteins were weakly acidic, because their average theoretical isoelectric point (pI) was 6.48. Only the instability index of CmbHLH42 protein was less than 40, and only the grand average of hydropathicity (GRAVY) value of CmbHLH49 protein was greater than zero, which meant that most CmbHLH proteins were unstable and hydrophilic. The aliphatic index of CmbHLH proteins varied from 49.33 to 109.53. A total of 6,831 microRNA (miRNA) targets were identified in *CmbHLHs*, indicating that corresponding miRNAs can activate or inhibit gene expression (Supplementary Table S3). In addition, 180 simple sequence repeats (SSRs) were predicted in the *CmbHLHs*, and the 3D structure models of CmbHLH proteins were predicted (Supplementary Tables S4, S5; Supplementary Figure S1). All these results provided valuable information reference for further research on the *CmbHLH* genes.

The classification of plant bHLH proteins had always been vague, but it was generally considered to be divided into 15–32 subgroups (Pires and Dolan, 2010b; Carretero-Paulet et al., 2010). We used the maximum likelihood method to construct an unrooted phylogenetic tree to explore the evolutionary relationships of *CmbHLH* genes (Figure 1B). The 94 CmbHLH proteins were classified into 19 subgroups based on the unrooted tree, which were named A to S (Figure 1B). Interestingly, subgroup J and K contained a single CmbHLH protein (CmbHLH16 and CmbHLH22), respectively. Except subgroup J and K, the number of *CmbHLH* genes in each subgroup varies greatly from 3 (subgroup D, H, Q and R) to 10 (subgroup E). In addition, we also constructed two phylogenetic tree of CmbHLH proteins with neighbor-joining and Bayesian method, respectively (Supplementary Figures S2, S3). The results showed that the phylogenetic trees of *CmbHLHs* constructed by the three methods were almost identical, with 94 *CmbHLHs* divided into 19 subgroups. Gene Ontology (GO) and The Kyoto Encyclopedia of Genes and Genomes (KEGG) enrichment analysis were performed to characterize the potential functions of the identified *CmbHLH* genes. As shown in Figures 1C, D, *CmbHLH* genes were mainly involved in RNA biosynthetic process, positive regulation of gene expression, and transcription regulator activity, and these functions and processes were closely related to transcription factors. KEGG enrichment results indicated that they were mainly enriched in transcription factors, signal transduction, plant hormone signal transduction, environmental information processing, which were the main mechanisms by which bHLH family transcription factors regulate the expression of downstream genes.

### Motif distribution, gene structure and promoter *cis*-elements analysis

**FIGURE 2:**
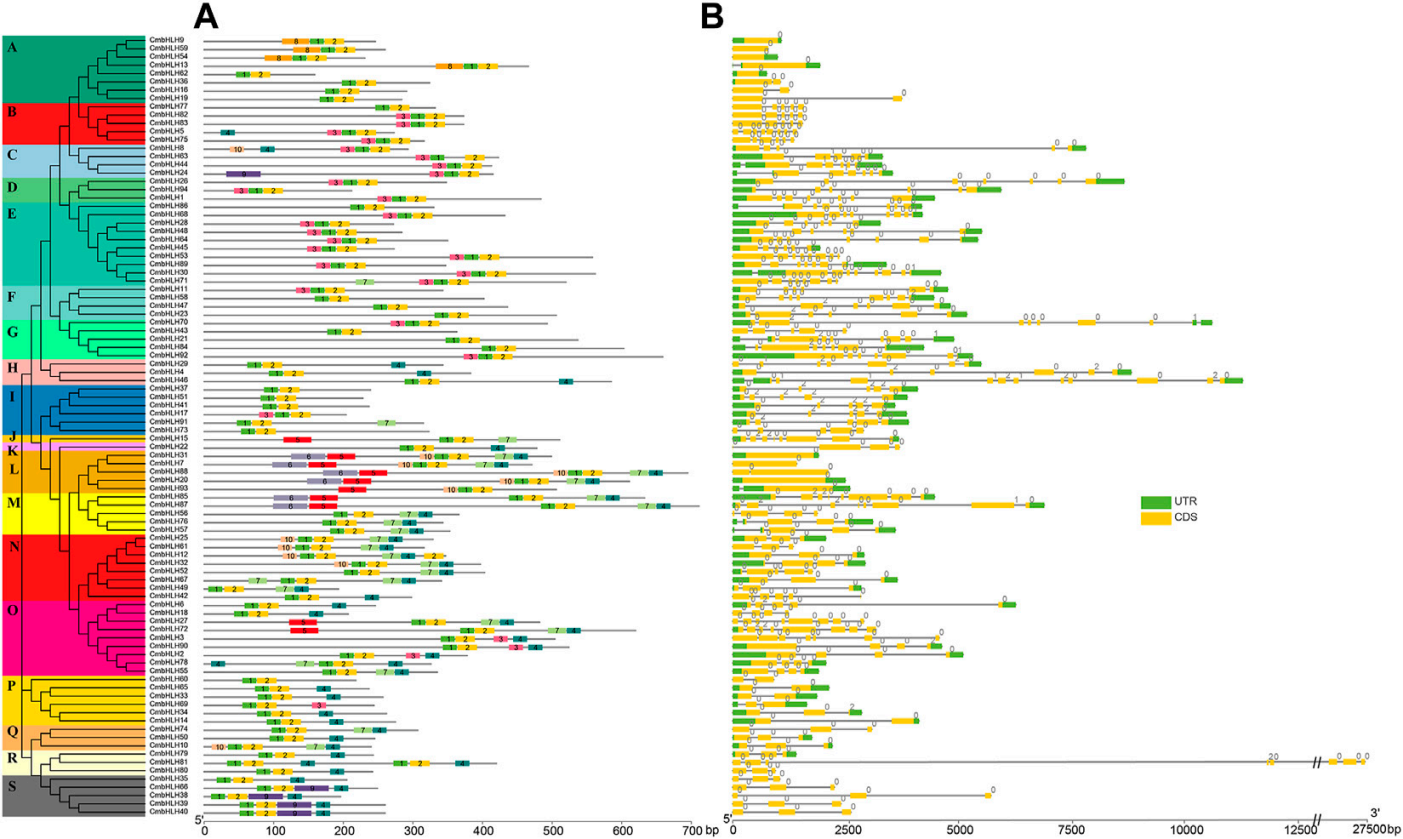
The conserved motifs distribution and gene structure of *CmbHLH* genes. **(A)** Distribution of conserved motifs of *CmbHLH* genes. The left side dendrogram shows the phylogenetic tree of *CmbHLH* genes. **(B)** Gene structure of *CmbHLH* genes.

The motif diversity and gene structure of *CmbHLH* genes were exhibited to reveal their evolution (Figure 2). The online MEME program was performed to identify motif patterns, and *CmbHLH* genes contained varying numbers of conserved motifs (Figure 2A). *CmbHLH* members in the same subgroup usually had similar motif arrangement patterns, while members in different subgroups had obvious differences. Although motifs 1 and 2 were present in almost all *CmbHLH* genes, some of the other motifs were detected in some specific subgroups. For example, motif 8 only can be detected in subgroup A, and motif 10 only can be detected in subgroup N. Interestingly, it was found that some *CmbHLH* genes in M subgroup had the same motif composition as the members in L subgroup, but some members of the M subgroup had completely different motif arrangements. This result implied that some *CmbHLH* genes had undergone dramatic changes in their non-motif sequences during evolution and formed the new subgroups without motif composition changing. Although the gene structure analysis indicated that there was extensive variation in the number of introns (0–11 introns) and gene structure of the *CmbHLH* genes, the members in the same subgroup had similar intron-exon structure (Figure 2B). For example, members in subgroup A had 0 or 1 intron, while members in subgroup Q had 3 introns. Interestingly, some *CmbHLH* members had exactly motif arrangement and extreme similar gene structure, such as *CmbHLH39* and *CmbHLH40*, and subsequent analysis showed that they were formed from tandem duplication. These results indicated that the members in the same subgroup had similar motifs arrangement pattern and gene structure, which suggested that they may have similar functions.

**FIGURE 3:**
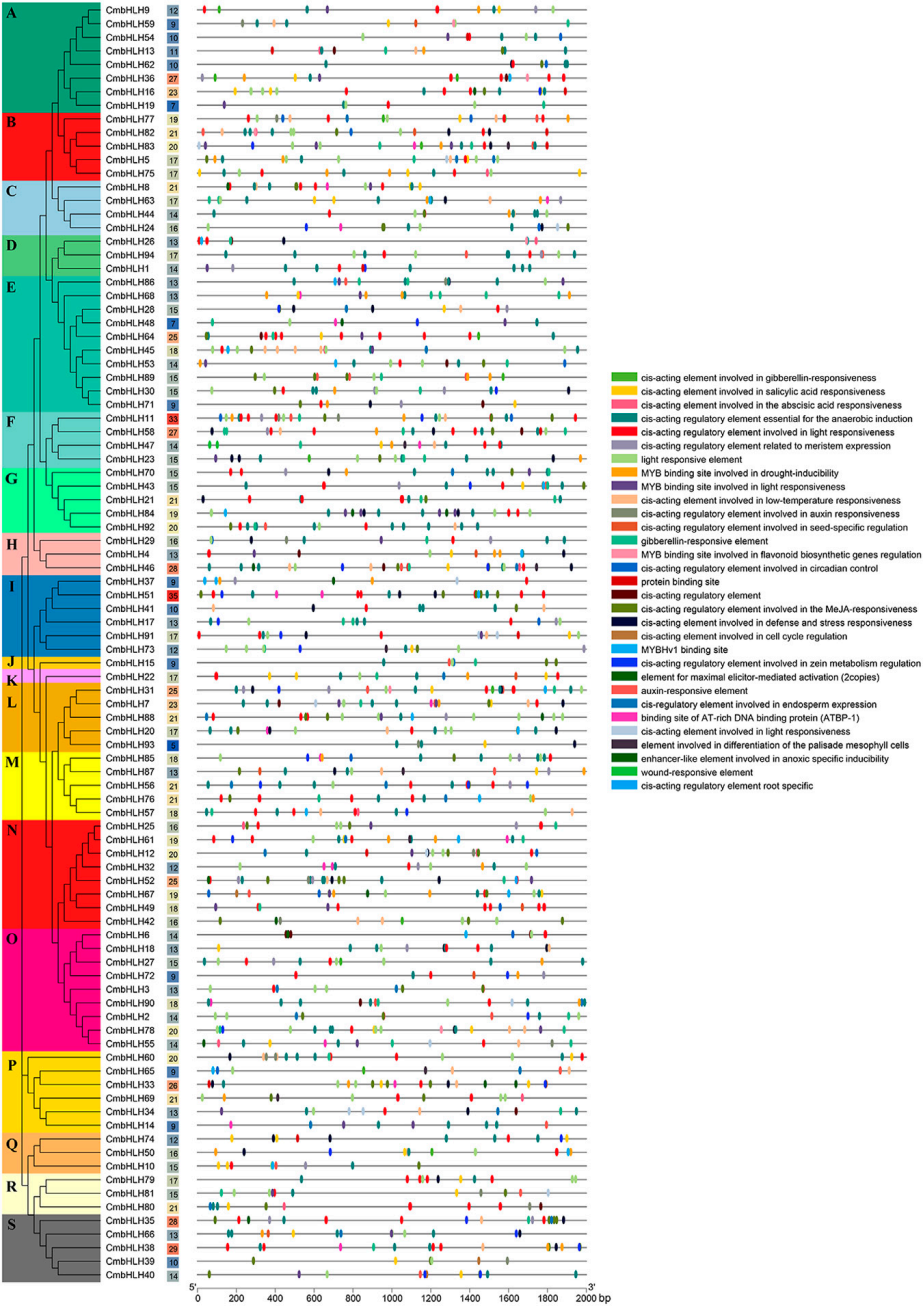
The *cis*-acting elements detected in the promoter regions of *CmbHLH* genes. The left side dendrogram shows the phylogenetic tree of *CmbHLH* genes. The number of *cis*-acting elements in the promoter region of each *CmbHLH* gene was shown in the middle of the figure. The distributions of *cis*-acting elements in the 2,000 bp upstream promoter are shown. The different functions of *cis*-acting elements are represented by different colors, as shown on the right.

The upstream 2,000 bp sequences of the *CmbHLH* genes were extracted to predict *cis*-acting elements in promoter regions, which can further explore the potential function and regulatory mechanism of the *CmbHLHs* (Figure 3; Supplementary Table S6). A total of 1,562 *cis*-acting elements were detected in promoter regions of *CmbHLHs* by the PlantCare software. Abundant *cis*-acting regulatory elements related to endosperm expression, meristem expression, and responses to gibberellin (GA) and auxin were identified in the upstream sequences of *CmbHLH* genes. This indicated that these genes may have potential functions in the morphogenesis of chestnut. Additionally, numerous *cis*-acting elements related to environment and stress were detected, such as *cis*-acting element involved in defense and stress responsiveness, *cis*-acting element involved in low-temperature responsiveness, *cis*-acting regulatory element essential for the anaerobic induction, and wound-responsive element. Notably, the *cis*-acting elements commonly considered important in relation to hormone response were detected, such as auxin-responsive element, gibberellin-responsive element, and salicylic acid responsive element, which were believed to be widely involved in all aspects of plant growth and development (Woodward and Bartel, 2005; Yamaguchi, 2008; Peng et al., 2021). The *cis*-acting elements involved in many functions were identified, which suggested that *CmbHLH* genes played diverse roles in chestnut.

### Collinearity analysis of *CmbHLHs*

**FIGURE 4:**
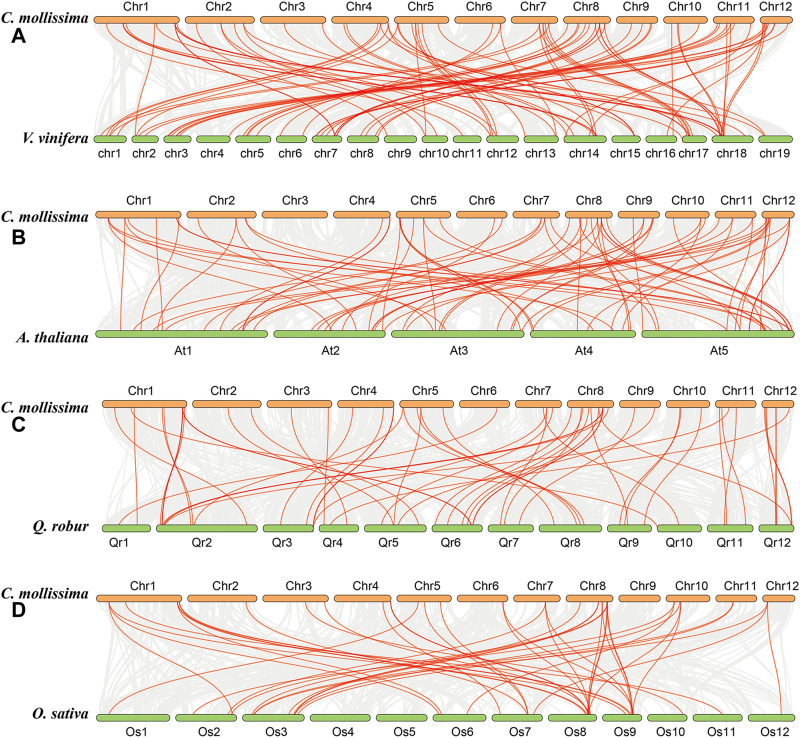
The collinearity among chestnut, grape, *Arabidopsis thaliana*, oak, and rice genomes obtained through MCScanX. **(A)** Collinearity relationship between chestnut and grape genomes. **(B)** Collinearity relationship between chestnut and *Arabidopsis thaliana* genomes. **(C)** Collinearity relationship between chestnut and oak genomes. **(D)** Collinearity relationship between chestnut and rice genomes.

The collinearity analysis among chestnut, grape, *A. thaliana*, oak, and rice genomes was performed to further investigate the evolution of the *CmbHLH* genes (Figure 4). Interestingly, we detected 68 *CmbHLHs* in the collinearity regions of the chestnut and grape genomes, and there were only 51, 50 and 25 *CmbHLHs* in the collinear regions between chestnut and *A. thaliana*, chestnut and oak, chestnut and rice, respectively (Supplementary Tables S7–S10). Additionally, there were 88 orthologous gene pairs contained *CmbHLHs* between chestnut and grape genome, and only 74, 57 and 44 orthologous gene pairs were found between chestnut and *A. thaliana*, chestnut and oak, chestnut and rice, respectively (Supplementary Tables S7–S10). As we all know, grape had a relatively stable genome structure, which was often used as a good reference for understanding the genome or related gene evolution of other eudicot plants (Consortium et al., 2012; Yu et al., 2022a). Therefore, the chestnut and grape genomes had not experienced additional whole genome duplication (WGD) events after the eudicot common hexaploidization event (ECH) (Yu et al., 2022b; Hu et al., 2022), and the grape genome had a relatively stable genomic structure, which resulted in the above statistical results about chestnut and grape. The distant genetic relationship between chestnut and rice, and rice genome had experienced additional WGD after ECH, may result in the least *CmbHLHs* in their collinear regions, and the least orthologous gene pairs contained *CmbHLHs* between them.

Significantly, we can detect some *CmbHLHs* existed in three or more collinear gene pairs, such as *CmbHLH1*, which suggesting that more copies of their orthologous genes in *A. thaliana*, rice, oak and grape genomes had been retained (Figure 4). According to the gene balance hypothesis, the products of these *CmbHLHs* may participate in macromolecular complexes or signaling networks (Birchler and Veitia, 2007). The subsequent transcriptome analysis revealed that they may have important functions in the bud development and ovule fertility of chestnut. Additionally, the collinear blocks contained *CmbHLHs* generally including more gene pairs in chestnut and grape (average of 49 gene pairs) (Supplementary Table S11). Contrasty, there were only 19, 27 and 11 (average value) gene pairs per block contained *CmbHLHs* between chestnut and *A. thaliana*, between chestnut and oak, between chestnut and rice, respectively (Supplementary Tables S12–S14). We speculated that the differences in WGD experience and genetic relationships of these species may be the cause of these results.

**FIGURE 5:**
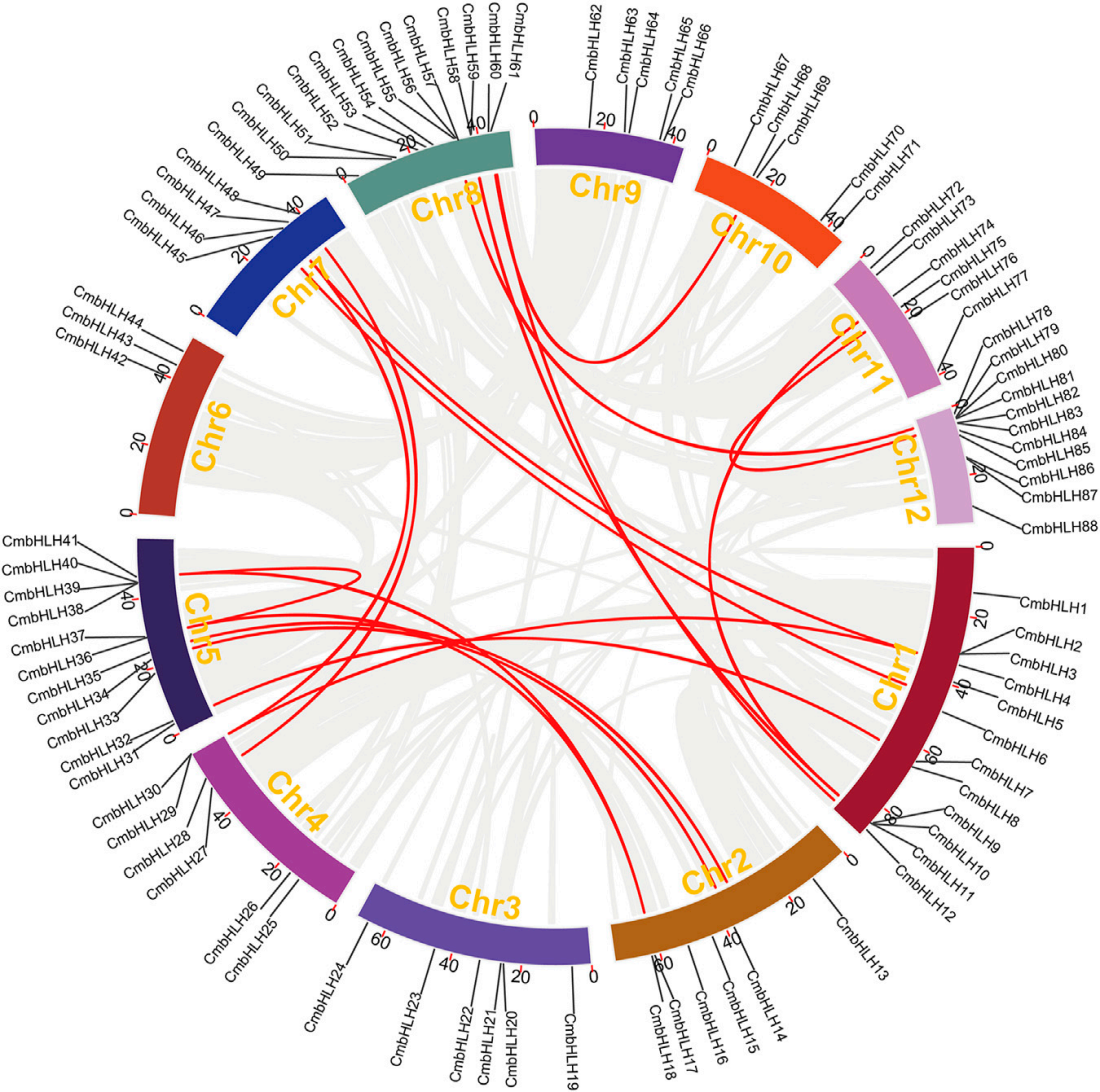
Circos-plot showing the collinearity of the *CmbHLH* genes.

**FIGURE 6:**
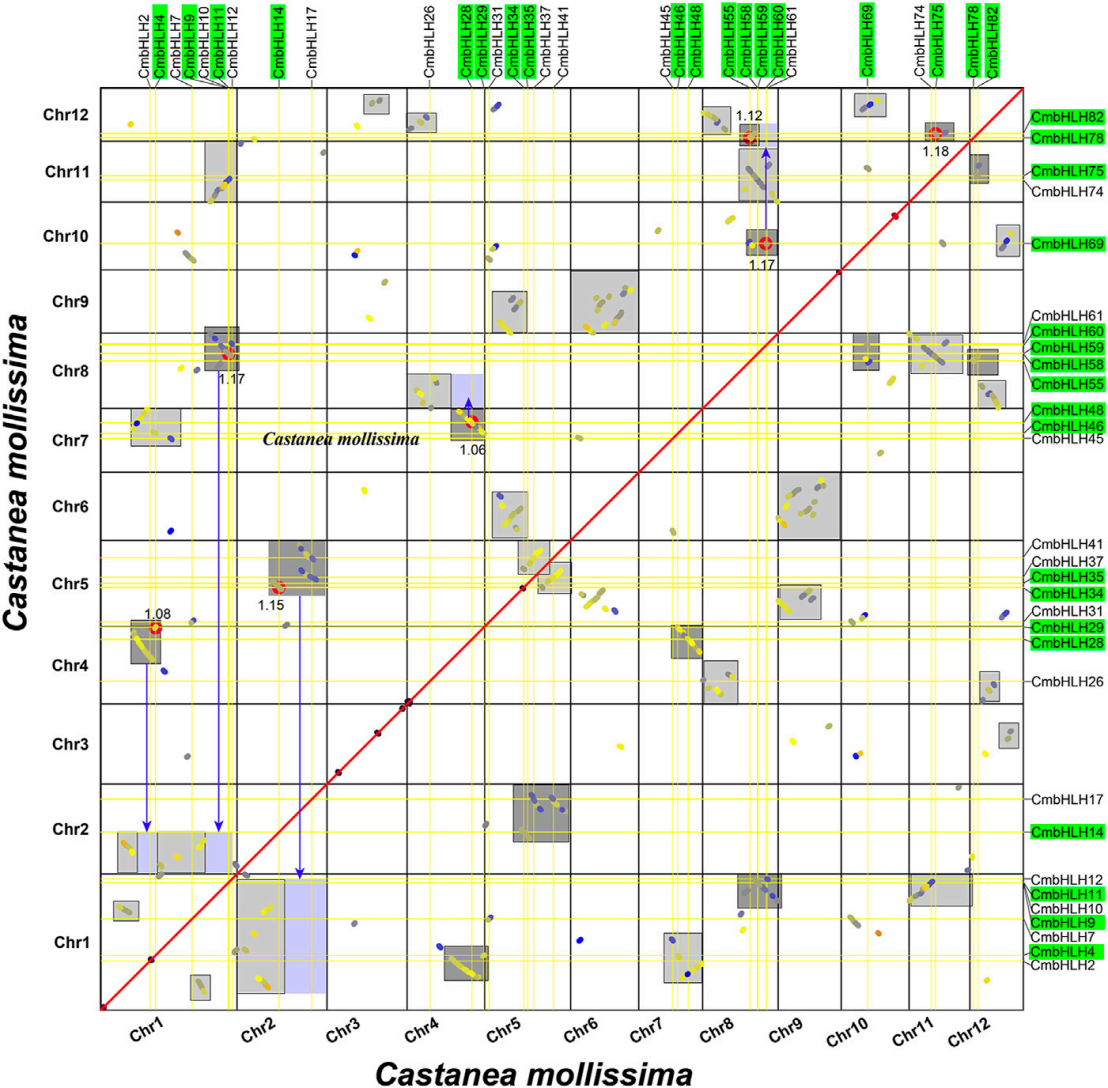
Homologous collinearity dot-plot within chestnut genome. The collinear blocks from WGD containing *CmbHLH* genes are marked in the gray boxes of the figure, and the median Ks of the collinear blocks are marked.

Gene duplication event was an important motivation for the expansion of gene families and the occurrence of new functions (Liu et al., 2018; Quan et al., 2019; Yu et al., 2021). The Multiple Collinearity Scan toolkit (MCScanX) program (Wang et al., 2012) and writing script were used to conduct collinearity analysis of chestnut genome to determine the duplication model of *CmbHLHs* (Yu et al., 2021). MCScanX could identify most duplication types except WGD and segmental duplication, and the reason was that the plant genome undergone a large number of chromosome recombination after WGD, which made them difficult to distinguish (Figure 5) (Wang et al., 2018; Yu et al., 2022a; Wang et al., 2022; Yu et al., 2023). As in our previous study (Yu et al., 2023), by drawing the homologous collinearity dot-plot within chestnut genome labeled with the median Ks of the homologous blocks, combining their length and complementarity, WGD and segmental duplications were finally distinguished (Figure 6). The results indicated that most of the *CmbHLHs* formed from dispersed duplication, followed by WGD, and no *CmbHLH* member was considered as singletons (Supplementary Table S15). These results showed that dispersed duplication was the main reason for the increase in the member of the bHLH gene family in chestnut. Additionally, Ka/Ks values of homologous gene pairs were calculated, which indicated that all of *CmbHLHs* were experienced purifying selection pressure during evolution (Ka/Ks < 1) (Supplementary Table S16).

### Expression analysis of *CmbHLHs*

**FIGURE 7:**
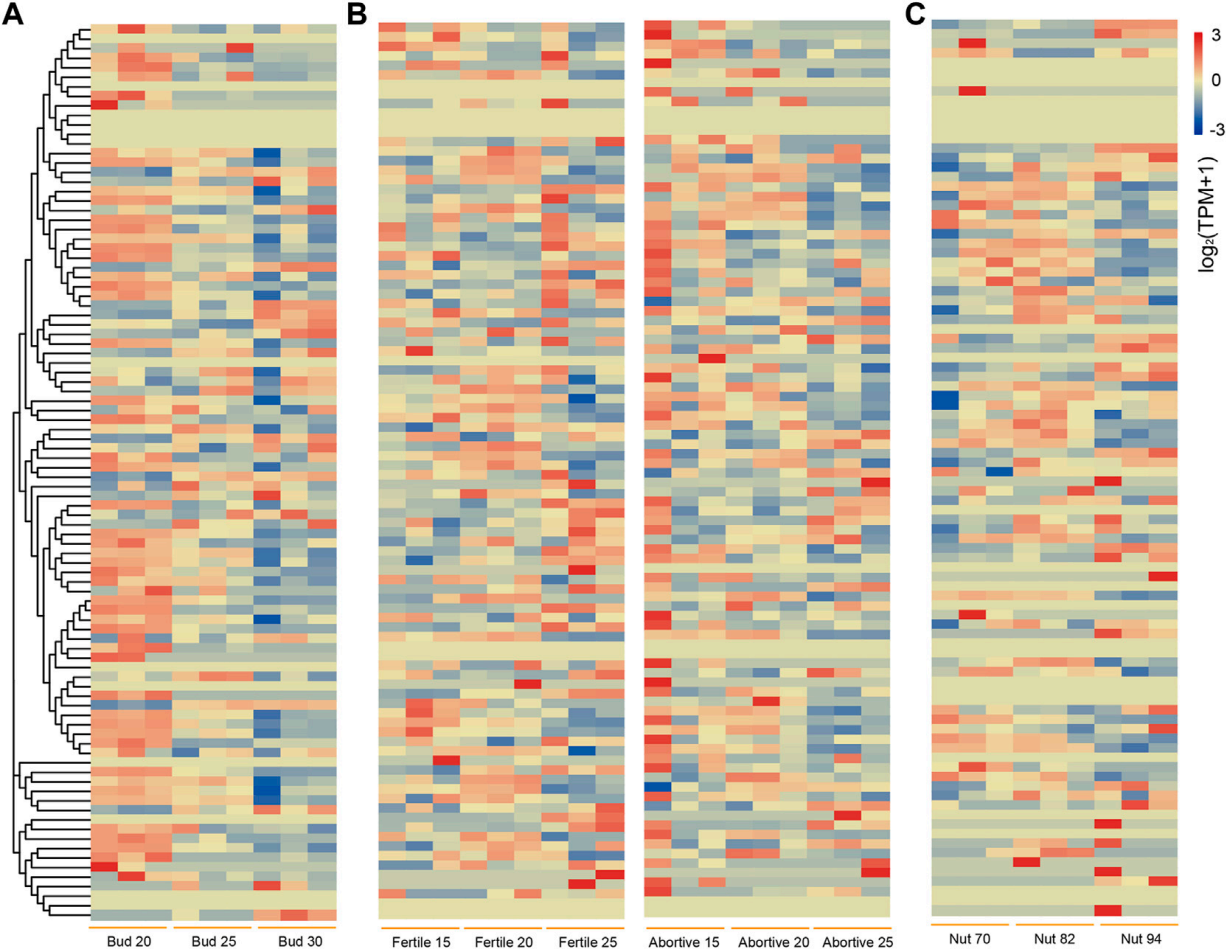
Heatmaps of *bHLH* genes expression in different stages of chestnut buds, nuts, fertile/abortive ovules. **(A)** Heatmap of *bHLH* genes expression in chestnut buds at 20, 25 and 30 days post-anthesis. The left side dendrogram shows the phylogenetic tree of *CmbHLH* genes. **(B)** Heatmap of *bHLH* genes expression in fertile and abortive ovules of chestnut on 15-July, 20-July and 25-July. **(C)** Heatmap of *bHLH* genes expression in chestnut nuts at 70, 82 and 94 days post-anthesis.

The transcriptome data of chestnut buds at different stages from NCBI were investigated to explore their potential function in chestnut bud development (Figure 7A). Some *CmbHLH* genes had high expression level at the all stages of chestnut bud development, suggesting their potential function in the chestnut bud development process. For example, the average TPM values of *CmbHLH88* were 138.16, 165.03 and 155.20 at three stages, respectively. Notably, the expression level of some *CmbHLH* genes decreased (*CmbHLH25*) or increased (*CmGRAS51*) sharply as the buds continue to growth. The TPM value of *CmbHLH25* in chestnut buds at 20 days post-anthesis was 107.91, while the TPM values at 25 and 30 days post-anthesis were 27.07 and 3.44, respectively. The TPM value of *CmbHLH51* in chestnut buds at 20 days post-anthesis was 34.37, while the TPM values at 25 and 30 days post-anthesis were 71.61 and 109.33, respectively. These results indicated that *CmbHLHs* play an important role in chestnut bud development.

In addition, we analyzed transcriptome data from fertile/abortive ovules of chestnut at different developmental stages to characterize the possible role of the *CmbHLH* genes in ovule fertility (Figure 7B) (Du et al., 2021). We found that the expression levels of a few *CmbHLHs* in fertile/abortive ovules showed significant differences, such as *CmbHLH55* and *CmbHLH69.* The expression level (TPM of all samples was more than 200) of *CmbHLH55* in fertile ovules was significantly higher than that in abortive ovules at all three stages, while the expression of *CmbHLH69* of fertile ovule (TPM of all samples were less than 50) was significantly lower than that of abortive ovules at all three stages. Additionally, the expression levels of some *CmbHLH* members had significantly changed during the growth of fertile ovules. For example, the TPM value of *CmbHLH69* on 15-July was 86.24, while the TPM values on 20-July and 25-July reached 151.44 and 144.10, respectively. Notably, multitudinous of gibberellin (GA) responsive elements had been detected in their promoter regions, and GA was considered to be a plant hormone that was extremely important for plant reproductive organs (Figure 3) (Plackett et al., 2014). *Cis*-regulatory element involved in endosperm expression had also been identified in the promoter regions of these *CmbHLHs*, which indicated that they may have impact on the fertility or development of chestnut ovules.

**FIGURE 8:**
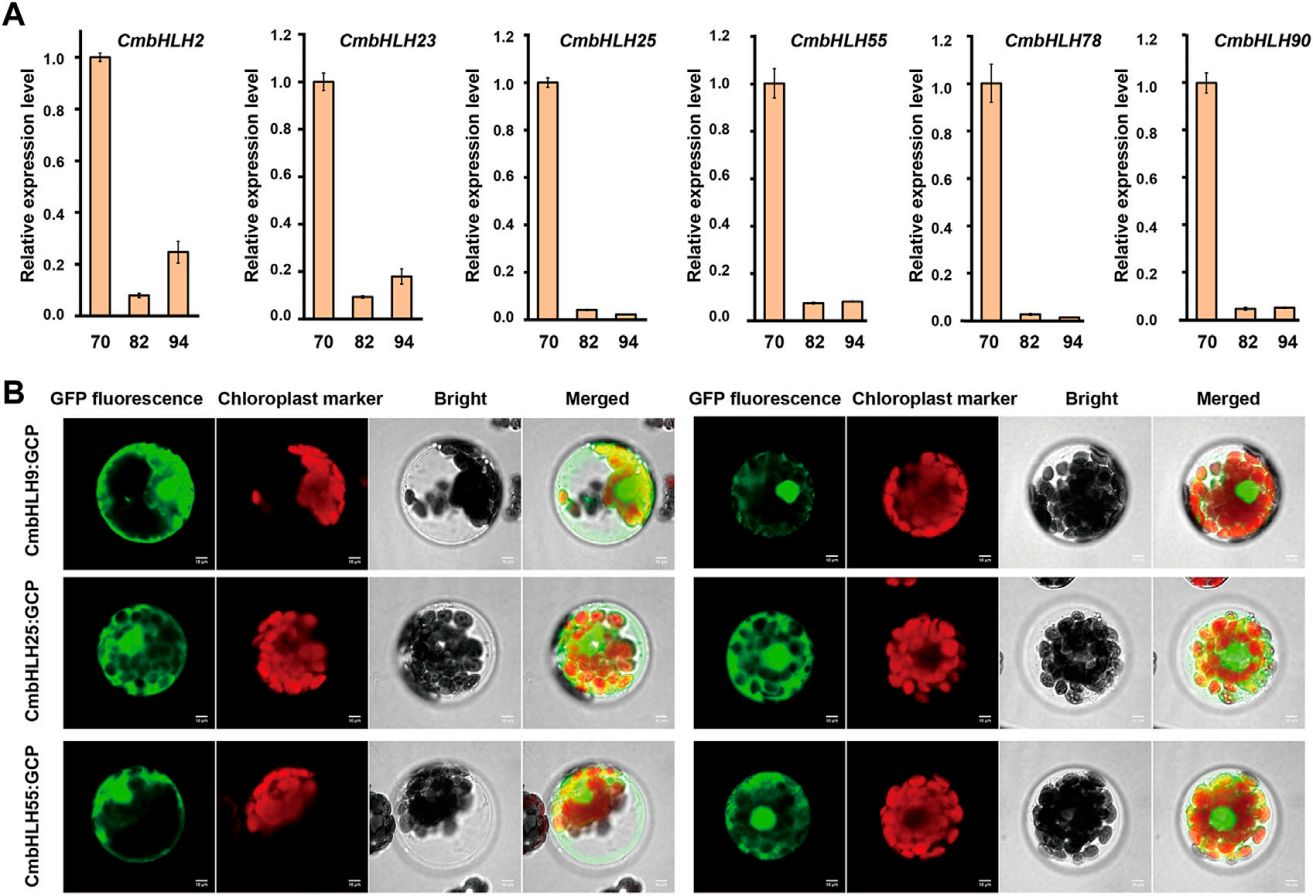
qRT-PCR of six *CmbHLHs* (*CmbHLH2*, *CmbHLH23*, *CmbHLH25*, *CmbHLH55*, *CmbHLH78* and *CmbHLH90*) in chestnut nuts at different developmental stages and subcellular location of three *CmbHLHs* (*CmbHLH9*, *CmbHLH25* and *CmbHLH55*) proteins in *Arabidopsis thaliana* protoplasts. **(A)** qRT-PCR of *CmbHLH2*, *CmbHLH23*, *CmbHLH25*, *CmbHLH55*, *CmbHLH78* and *CmbHLH90* in chestnut nuts at different developmental stages. **(B)** Subcellular location of *CmbHLH9*, *CmbHLH25*, *CmbHLH55* proteins in *Arabidopsis thaliana* protoplasts.

Similarly, transcriptome data from chestnut nuts at different developmental stages were analyzed (Figure 7C) (Li et al., 2021). Obviously, the expression of some *CmbHLH* genes at 94 days post-anthesis of chestnut nut development had reduced dramatically, such as *CmbHLH25* and *CmbHLH78*. For example, the average TPM values of *CmbHLH25* were 274.67 at 70 days post-anthesis, and this values were 164.14 and 1.29 at 82 and 94 days post-anthesis, respectively. Considering that some *bHLH* genes had been verified to be involved in the regulation of secondary metabolite formation (Li et al., 2006) and that some *cis*-acting regulatory elements involved in the regulation of zein metabolism and flavonoid biosynthesis had been discovered (Figure 3; Supplementary Table S6), we believed that these *CmbHLH* members may be related to chestnut nuts ripening and related quality. To verify the expression patterns of *CmbHLHs*, six *CmbHLH* genes were analyzed using qRT-PCR, and the results were shown in Figure 8A. Overall, the qRT-PCR experiments analysis results were consistent with the RNA-seq analysis. For example, *CmbHLH25* was significantly highly expressed in nuts at 70 days post-anthesis than in nuts at 82 and 94 days post-anthesis. The expression level of *CmbHLH2* was highest at 70 days post-anthesis, and decreased to the lowest at 82 days post-anthesis, and increased at 94 days post-anthesis. The expression trends of other four *CmbHLH* genes analyzed using qRT-PCR during chestnut nut development were also consistent with the results of RNA-seq analysis. In addition, almost all CmbHLH proteins (85/94) were predicted in the nucleus, based on subcellular localization analysis (Supplementary Table S2). We instantaneously expressed GFP-CmbHLH9, GFP-CmbHLH25, and GFP-CmbHLH55 fusion proteins in *A. thaliana* protoplasts for subcellular localization. As shown in Figure 8B, *CmbHLH25* and *CmbHLH55* were co-localized to both the nucleus and cytoplasm, and *CmbHLH9* was localized to the nucleus.

## Discussion

Numerous studies have shown that the bHLH gene family had been formed in algae, but their number was relatively small, and the bHLH domain had not undergone significant differentiation (Robinson and Lopes, 2000; Pires and Dolan, 2010a; Fan et al., 2021; Song et al., 2021). Accordingly, the function of the *bHLH* genes in algae may be limited, and the number of downstream genes that can be regulated was also small (Robinson and Lopes, 2000; Pires and Dolan, 2010a). In the process of algae evolving into various complex plants, the number of members in bHLH gene family had greatly expanded, indicating the important role of the *bHLH* gene function in plant evolution (Kurbidaeva et al., 2014). The current functional characterization of the *bHLH* genes in many higher plants indicates that they have important functions in various biological processes such as morphogenesis, stress resistance, signal transduction, and secondary metabolism (Li et al., 2006).

The number and classification of *bHLH* genes in plants showed great diversity. For example, there were 161, 167, and 152 *bHLH* genes in *A. thaliana*, rice, and tomato, and they were divided into 21, 22, and 24 subgroups, respectively (Sun et al., 2015). In this study, we identified 94 *bHLH* genes in the chestnut genome and divided them into 19 subgroups using three methods (the neighbour-joining, maximum likelihood, and Bayesian methods). Similar gene structures were found in *CmbHLHs* within the same subgroup, while members belonging to the same subgroup showed differences in motif composition (Figure 2). For example, *CmbHLH77* lacked a motif 3 compared to other members in the same subgroup B, and the motif composition of *CmbHLH77* was completely consistent with most members of subgroup A. These results suggested that *CmbHLH77* may loss motif 3 during evolution, but retained other features of members in subgroup B. Considering that gene duplication was an important driving force for the expansion of many gene families (Yu et al., 2022b; Yu et al., 2023), we in-depth analyzed the duplication models of the *CmbHLH* genes. Notably, previous studies on gene duplication had rarely distinguished WGD from segmental duplication, due to the large number of gene losses and chromosome fusion that accompany WGD in plant genomes (Yu et al., 2022a). Here, we conducted combined analyses of the collinear blocks of the chestnut genome and the Ks that can characterize the time of gene duplication, as in our previous study (Yu et al., 2022a). Grossly, the collinear regions formed by WGD were usually larger blocks (covering more gene pairs), and the Ks values of gene pairs on that collinear blocks were similar, corresponding to the Ks value at the time of the WGD event (Supplementary Table S16). Based on the above principles, we distinguished the duplication models of all *CmbHLH* genes, and the results showed that dispersed duplication and WGD were the main reasons for the expansion of the *CmbHLH* genes, which highlighted the importance of transposable elements and polyploidization in the expansion of chestnut bHLH gene family. WGD is one of the important origins of early plant genes, but the mechanism of dispersed duplication is still unclear (Bai et al., 2022). However, transposable elements (TEs) are considered the most common source of dispersed duplication (Kroon et al., 2016). Transposable elements have the flexibility to move their positions within the genome and play an important role in shaping the fate of gene function (Fueyo et al., 2022). In this study, a large number of dispersed duplication genes showed significant diversity in gene structure and expression patterns (Figures 2, 7). All the above analysis suggested that dispersed duplication may be one of the important reasons for the expansion and functional differentiation of the chestnut *bHLH* genes.

The *bHLH* genes played diverse roles in plant, especially in plant meristem and morphological development (Heisler et al., 2001; Bernhardt et al., 2005). *LAX PANICLE* (*LAX*), a *bHLH* gene in rice, was a major regulatory factor controlling the apical meristem of rice, and ectopic *LAX* expression in rice resulted in dwarfing (Komatsu et al., 2003). The *SPATULA* gene was first found in *A. thaliana* to participate in the control of the peripheral region of stem tip meristem and the development of specific tissues in leaves, petals, stigmas, and roots (Heisler et al., 2001). In this study, some *CmbHLH* genes that may have functions in the development of chestnut buds and fertile/abortive ovules were discovered based on analysis of transcriptome data. *CmbHLH88* was highly expressed at all stages of chestnut bud development (TPM >130 in examples of all stages), and *cis*-regulatory elements related to meristem expression had been detected in the *CmbHLH88* promoter region. These results prompted us to speculate that *CmbHLH88* may be involved in the chestnut buds development. Furthermore, the *bHLH* genes had also been found to participate in the formation of secondary metabolism in plants (Zhang et al., 2011; Nims et al., 2015). *OsbHLH148* in rice was involved in the jasmonic acid (JAs) signaling pathway, and JAs was an important hormone involved in regulating secondary metabolic biosynthesis in plants (Seo et al., 2011). It was found that the expression of some *CmbHLH* genes increased sharply during the late ripening stage (94 days post-anthesis) of chestnut nuts, such as *CmbHLH9*, and *cis*-acting regulatory elements involved in the regulation of zein metabolism and flavonoid biosynthesis in its promoter region were identified, which suggested that it may have potential functions for chestnut ripening.

Notably, the involvement of the *bHLH* genes in plant embryos has received widespread attention. *RGE1* had been proven to control the normal growth of embryos (Kondou et al., 2008). *PIF4* plays a role in the elongation of the hypocotyl of *A. thaliana* by participating in phyB-regulated photomorphogenesis (Huq and Quail, 2002). The *SPATULA* gene can ultimately affect the formation of pistils by promoting the growth of carpels (Heisler et al., 2001). Here, interesting phenomena were found in the transcriptome of ovules. The expression level of *CmbHLH55* in fertile ovules was significantly higher than that in abortive ovules during all developmental stages. Contrasty, the expression of *CmbHLH69* in fertile ovule was significantly lower than that of abortive ovules during all developmental stages. Additionally, multitude researchers had reported that gibberellin has important function in plant reproductive organs, especially in gender determination of flowers (Song et al., 2013; Yu et al., 2023). The large ratio of female and male flowers (1:2,400–1:4,000) severely consumes tree nutrients and limits the number of female flowers, which was the key reason for limiting chestnut production (Chen et al., 2019). Due to the gibberellin responsive elements (GRE) (Figure 3) identified in these *CmbHLH* genes promoter regions, and the significant differences in the expression of these genes in fertile/abortive ovules, we speculated that they may have a significant potential role in the fertility of chestnut ovules.

The important function of *bHLH* genes in plant was continuously being proven, such as GA signal transduction regulation, secondary metabolite formation and stress resistance, and these functions all may be used to lift restrictions on chestnut yield and quality (Li et al., 2006; Liu et al., 2015; Chen et al., 2019). Paying attention to the potential function of *bHLH* genes in plants was of great significance for improving the yield and quality of chestnut nuts, as well as the development of the entire chestnut industry. Here, the physicochemical properties, *cis*-acting elements, phylogenetic analysis, gene structure and duplication model of 94 *CmbHLH* genes had been comprehensively characterized in chestnut. Transcriptome data, qRT-PCR and subcellular localization were used to analyze the expression patterns of *CmbHLHs* in different development stages of chestnut buds, nuts, fertile/sterile ovules, and revealed some members may have potential functions in chestnut tissue development and nut quality. This study provides a reference for elucidating the *CmbHLH* genes evolution and their potential functions in the development of chestnut buds, nuts, and fertile/abortive ovules.

## Data Availability

The original contributions presented in the study are included in the article/Supplementary Material, further inquiries can be directed to the corresponding author.

## Supplementary material

SUPPLEMENTARY FIGURE S1 Prediction of 3D structure of CmbHLH proteins by Swiss model.

SUPPLEMENTARY FIGURE S2 Phylogenetic tree of CmbHLH genes was constructed by neighbor-joining method.

SUPPLEMENTARY FIGURE S3 Phylogenetic tree of *CmbHLH* genes was constructed by Bayesian method.
