# Supplementary figures and images for: Genome-wide identification, evolution and expression profiles analysis of bHLH gene family in Castanea mollissima
Source: Front Genet. 2023 May 12;14:1193953. doi: 10.3389/fgene.2023.1193953 (PMC10213225; doi:10.3389/fgene.2023.1193953)

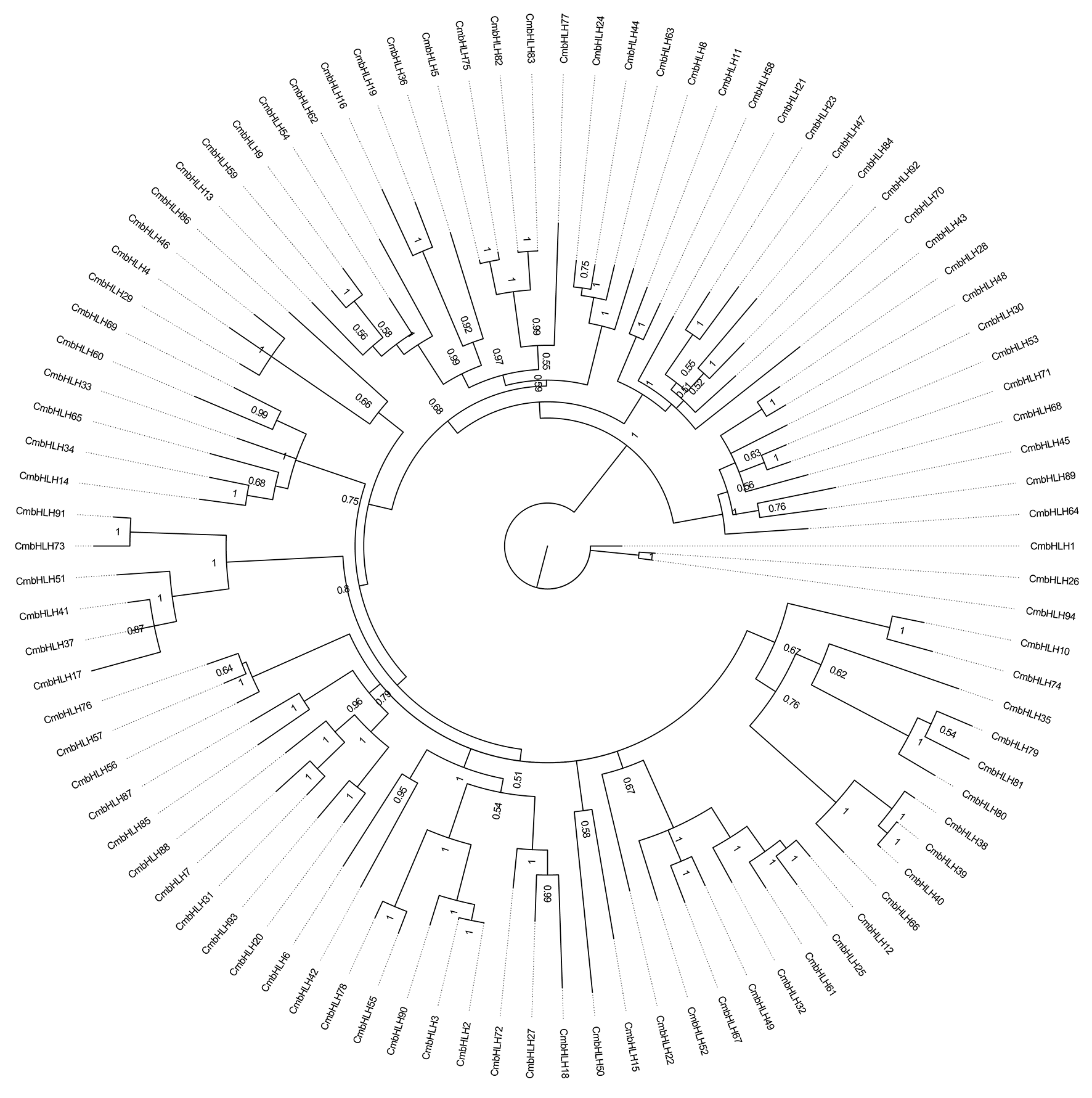

Supplement: Supplementary file 1 [file Image3.TIF]

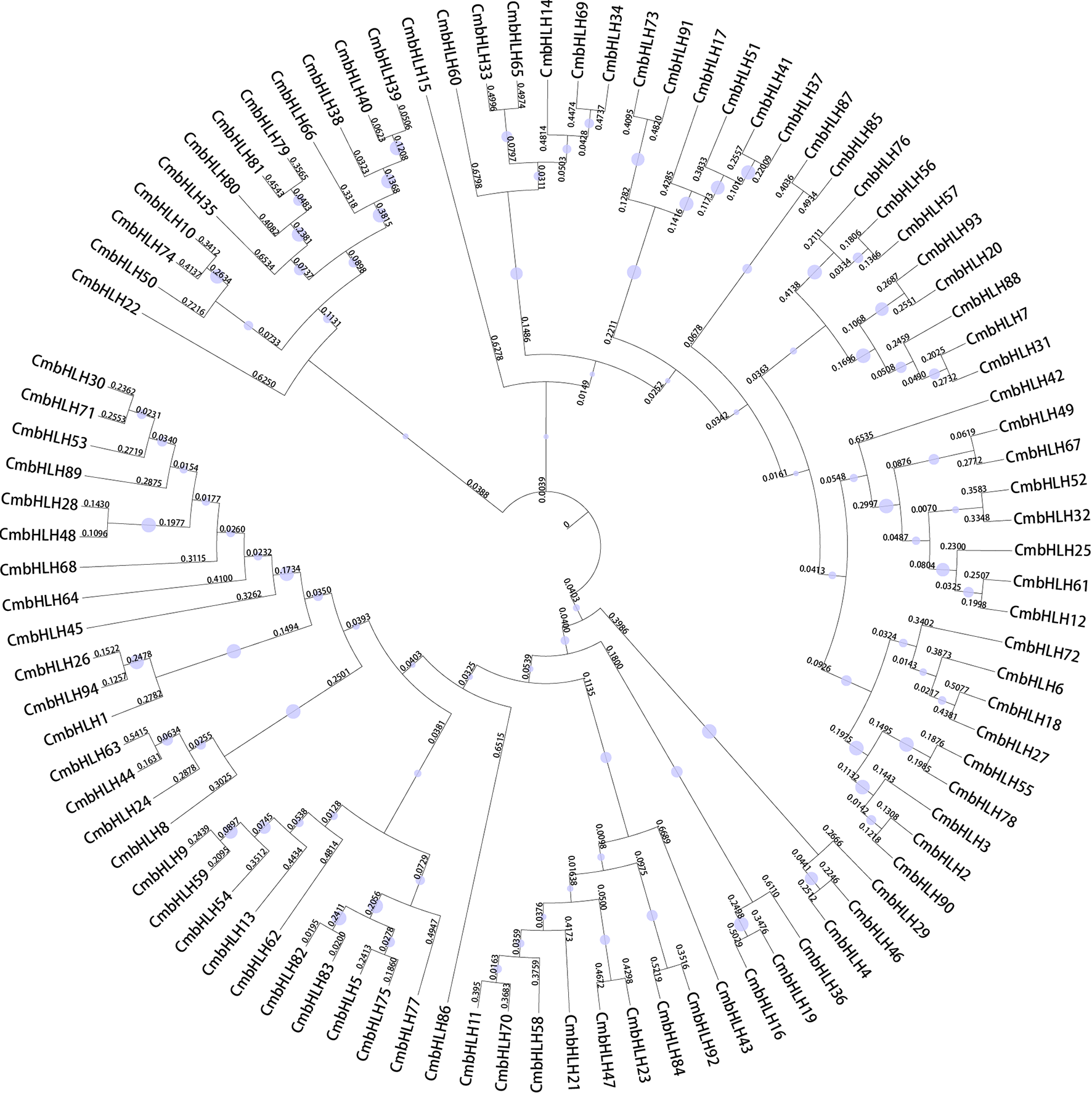

Supplement: Supplementary file 2 [file Image2.TIF]

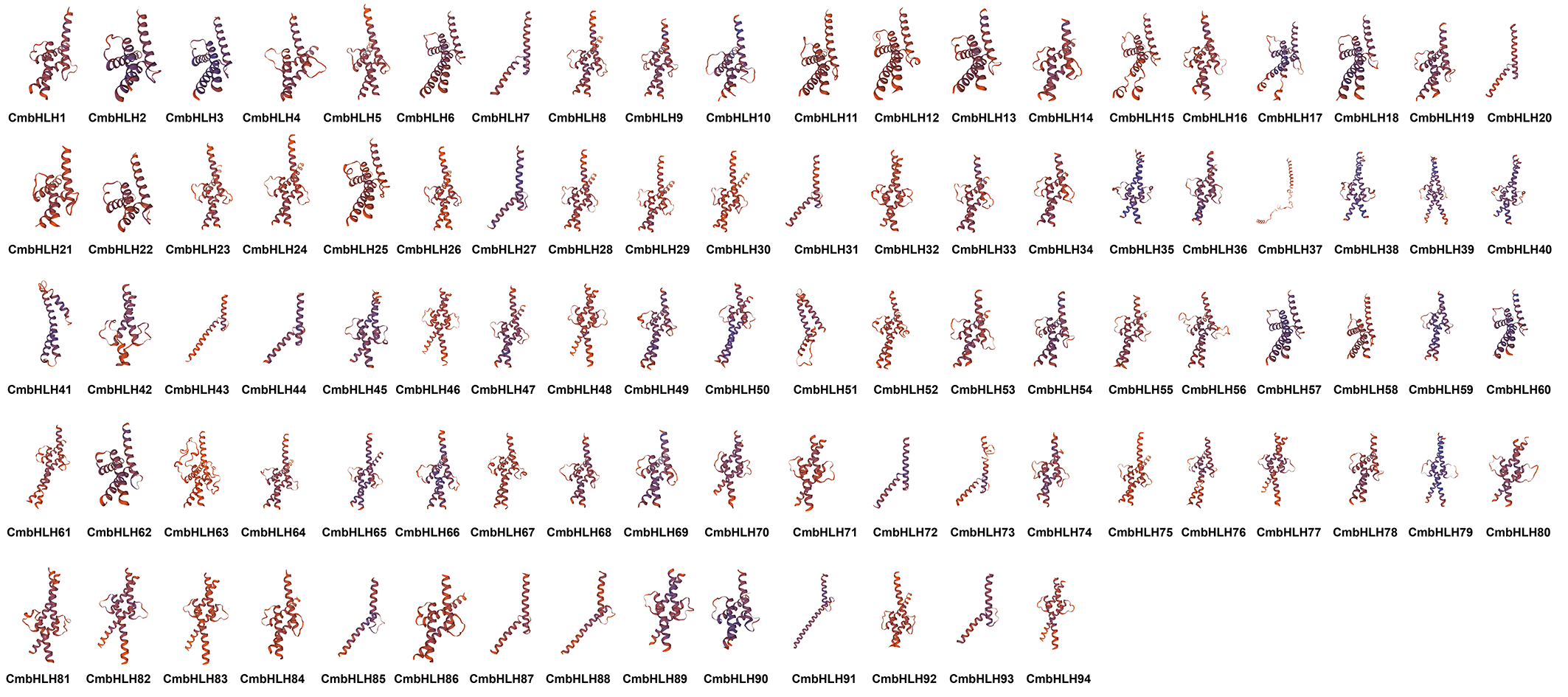

Supplement: Supplementary file 3 [file Image1.TIF]
